# Discovery and Validation of Hypermethylated Markers for Colorectal Cancer

**DOI:** 10.1155/2016/2192853

**Published:** 2016-07-14

**Authors:** Jiufeng Wei, Guodong Li, Shuwei Dang, Yuhui Zhou, Kai Zeng, Ming Liu

**Affiliations:** ^1^Department of General Surgery, The Fourth Affiliated Hospital of Harbin Medical University, Harbin 150001, China; ^2^Bio-Bank of Department of General Surgery, The Fourth Affiliated Hospital of Harbin Medical University, Harbin 150001, China

## Abstract

Colorectal carcinoma (CRC) is one of the most prevalent malignant tumors worldwide. Screening and early diagnosis are critical for the clinical management of this disease. DNA methylation changes have been regarded as promising biomarkers for CRC diagnosis. Here, we map DNA methylation profiling on CRC in six CRCs and paired normal samples using a 450 K bead array. Further analysis confirms the methylation status of candidates in two data sets from the Gene Expression Omnibus. Receiver operating characteristic (ROC) curves are calculated to determine the diagnostic performances. We identify 1549 differentially methylated regions (DMRs) showing differences in methylation between CRC and normal tissue. Two genes (*ADD2* and* AKR1B1*), related to the DMRs, are selected for further validation. ROC curves show that the areas under the curves of* ADD2* and* AKR1B1* are higher than that of* SEPT9*, which has been clinically used as a screening biomarker of CRC. Our data suggests that aberrant DNA methylation of* ADD2* and* AKR1B1* could be potential screening markers of CRC.

## 1. Introduction

Colorectal carcinoma (CRC) is one of the most prevalent malignant tumors worldwide. Global statistics showed that in 2012 alone, an estimated 1.36 million new cases were diagnosed with CRC, and approximately 694,000 people died from this disease [1]. Screening and early diagnosis are critical for the clinical management of CRC. Traditional screening tools include fecal occult blood test (FOBT) and colonoscopy. However, the effectiveness of FOBT is limited by the test performance, while colonoscopy is invasive, and it is therefore impractical to screen all patients for CRC in this manner. The identification of highly specific, noninvasive biomarkers is a top priority for screening and early diagnosis of CRC.

Aberrant DNA methylation is a well-recognized epigenetic feature of cancer, in general, and has been discovered in most tumors; it is thus gaining increasing attention as a potential biomarker [2–4]. Abnormally methylated genes can be used as biomarkers for early detection as well as tumor classification of CRC [5–7]. Some of these alterations have also been detected in stool or peripheral blood, suggesting that they can be candidates for noninvasive biomarkers of CRC. Epi proColon®, a blood-based assay for measuring methylated* SEPT9*, has become available for clinical application and has been approved by China and Europe. However, the sensitivity and specificity are still not satisfactory [5]. Novel biomarkers are needed to improve the accuracy of diagnosis of CRC.

In this study, the genome-wide methylation pattern of CRC was compared with adjacent normal tissues using the Illumina 450 K microarray, thus revealing aberrantly differentially methylated regions (DMRs) in CRC. Among the list of DMRs that we identified, potential biomarkers were validated in two independent data sets. We also established the sensitivity and specificity of the new molecular markers, which showed a higher area under the curve (AUC) than* SEPT9*. These biomarkers could improve the accuracy of CRC screening and diagnosis.

## 2. Materials and Methods

### 2.1. Subjects

Six pairs of CRC and adjacent normal tissues were obtained from the Bio-Bank of the Department of General Surgery of the Fourth Affiliated Hospital of Harbin Medical University. Inclusion criteria were no cancer other than CRC, no indications of heredity, and no radio- or chemotherapy prior to surgical resection. This study was approved by the Medical Research Ethics Committee of the Fourth Affiliated Hospital of Harbin Medical University, and informed consent was obtained. The diagnosis of CRC tissues was acquired from pathology reports. Fresh tissue samples were collected within 30 min after resection surgery, frozen in liquid nitrogen, and stored at −80°C. Clinicopathological characteristics of CRC patients are shown in Table S1 in Supplementary Material available online at http://dx.doi.org/10.1155/2016/2192853.

### 2.2. Human Methylation 450 K Microarray

Genomic DNA was extracted using standard phenol-chloroform techniques and quantified using Nanodrop 2000c. Genomic DNA from all samples was treated with an EZ Methylation Kit (Zymo Labs, Irvine, CA). Bisulfite-converted DNA (500 ng) was hybridized onto the Infinium Human Methylation 450 K BeadChip according to the manufacturer's standard protocol.

### 2.3. Differential Methylation Region Analysis

Infinium Methylation data were processed with the Methylation Module of the GenomeStudio software. Methylation levels of CpG sites were calculated as *β*-values (0-1). We removed unreliable probes that were detected with a* P* value > 0.05. In addition, CpG sites were removed on the* X* and* Y* chromosomes, containing single-nucleotide polymorphisms. The methylation data were deposited in the NCBI Gene Expression Omnibus (GEO): GSE75546. DMRs were analyzed using the ChAMP package, according to the instruction manual. To help identify regions of realistic length, the search was only conducted in regions where the distance between consecutive probes was less than 1 kb. The average *β*-values of the probes in the DMR were used as a representative of the DMR methylation levels. To screen the candidate DMR, the following criteria were used: *β*-difference > 0.4, *β*-value in normal tissue < 0.15, and *P*  value < 1*E* − 4.

### 2.4. Data Set for Validation of Candidate Biomarkers

Methylation of candidate markers was evaluated in the data sets GSE48684 (147 samples containing CRC, adenoma, and normal tissues) and GSE68060 (118 samples containing CRC and normal tissues) from the GEO. The methylation status of these samples was determined using the same version of the 450 K methylation array.

### 2.5. Statistical Analysis

Statistical analysis was conducted using the GraphPad Prism 6 software (La Jolla, CA, USA) and MedCalc version 10.1.6 (MedCalc Software, Mariakerke, Belgium). The Mann-Whitney *U* test was used to compare methylation levels between CRC, adenoma, and normal tissue. All reported *P* values were two-sided, with *P* < 0.05 being considered statistically significant. ROC analysis was performed by MedCalc.

## 3. Results

### 3.1. DMRs in Tumors versus Adjacent Normal Tissues

To identify DMRs related to colorectal carcinogenesis, whole genome DNA methylation analysis was performed using the ChAMP package with the Illumina 450 K bead array. Through this method, 1549 DMRs were identified with significant methylation differences between the six pairs of CRCs and adjacent normal samples. The top ten DMRs, according to *P* values, are shown in Table 1. A gene-based variant of the region-level test was performed, revealing 629 DMRs located in the promoter region, 207 DMRs located in the 5′ Untranslated Regions (UTR), 117 DMRs located at the 1st Exon, 592 DMRs located in the gene body, 99 DMRs located in the 3′UTR, and 841 DMRs located in the intergenic region. A CpG island-based variant of the region-level test was conducted in the same manner, revealing 391 DMRs distributed in CpG islands, 585 DMRs distributed within the shores, 347 DMRs distributed in the shelves, and 1162 DMRs distributed in the open sea (Table S2).

**Figure 1:**
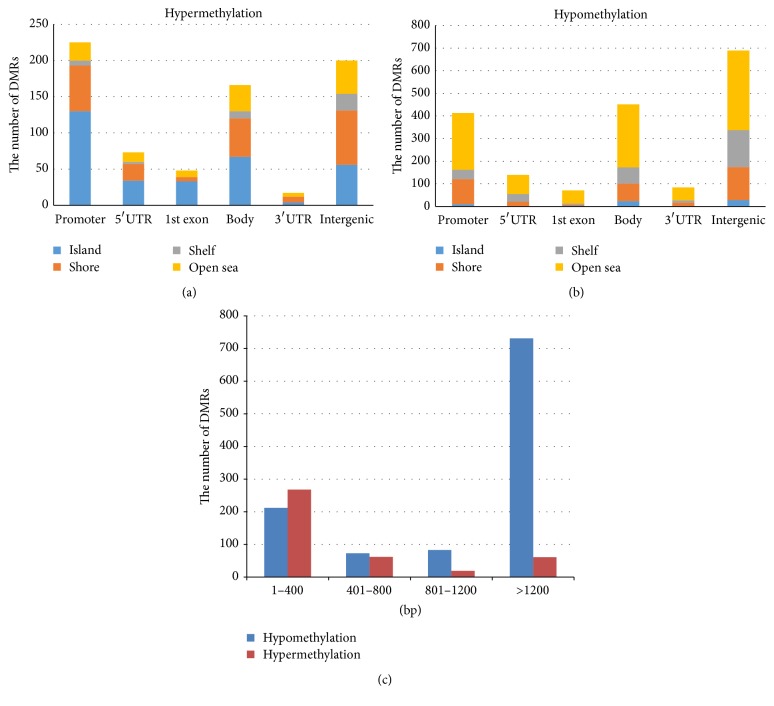
Distribution of DMRs in the human CRC genome. (a) Stacked bar charts showing the distribution of the hypermethylated DMRs over five gene categories: promoter, 5′UTR, 1st exon, gene body, 3′UTR, and intergenic regions. (b) Stacked bar charts showing the distribution of the hypomethylated DMRs over five gene categories: promoter, 5′UTR, 1st exon, gene body, 3′UTR, and intergenic regions. (c) Bar charts showing the distribution of the hypermethylated and hypomethylated DMRs considering four sizes: 1–400 bp, 401–800 bp, 801–1200 bp, and >1200 bp.

We calculated the hypermethylated and hypomethylated DMRs as shown in Figure 1. Hypermethylated DMRs were mainly located in the promoter region and CpG islands (Figure 1(a)), while hypomethylated DMRs were mainly located in the intergenic region and open sea (Figure 1(b)). Interestingly, most of the hypermethylated DMRs were less than 400 bp, but most of the hypomethylated DMRs were greater than 1200 bp (Figure 1(c)).

### 3.2. Identification of Candidate DNA Methylation Markers

To identify the candidate DNA methylation markers, the following criteria were used: *β*-difference > 0.4, *β*-value in normal tissue < 0.15, and *P*  value < 1*E* − 4. Identification was restricted to hypermethylated DMRs as these can be easily transferred to clinical application with Methylation-Specific Polymerase Chain Reaction (MSP). After evaluating all DMRs, three DMRs were identified that met all the criteria. Information regarding these three DMRs is shown in Table 2. Of these three DMRs-related genes, SEPT9 has been clinically used as a screening biomarker. In the present study, SEPT9 was used as a reference.

### 3.3. *In Silico* Validation of Selected Candidates

**Figure 2:**
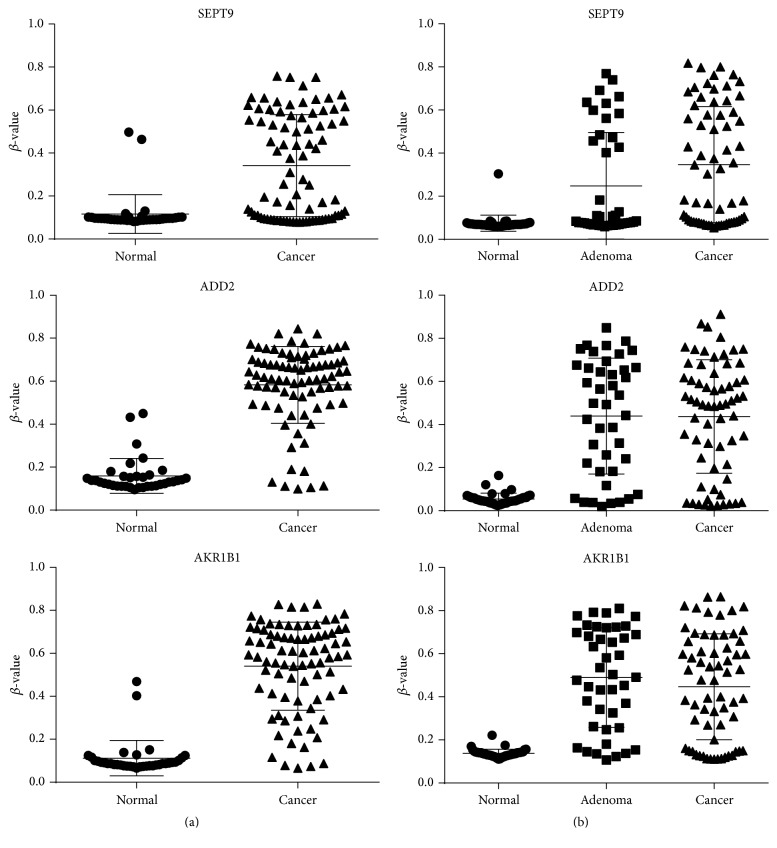
The different methylation levels of three genes in normal tissue, adenoma, and CRC. (a) The strip plot shows the different methylation levels of three genes (SEPT9, ADD2, and AKR1B1) in normal tissue and CRC with the data set GSE68060 from GEO. (b) The strip plot shows the different methylation level of three genes (SEPT9, ADD2, and AKR1B1) in normal tissue, adenoma, and CRC with the data set GSE48684 from GEO.

To investigate the selected DNA methylation candidates, we used two independent data sets, namely, GSE48684 and GSE68060, from GEO. GSE48684 contained 41 normal tissues, 42 adenomas, and 64 CRCs. GSE68060 contained 36 normal tissues and 82 CRCs. The data set GSE48684 was generated from the 450 K methylation array. Technical and biological validation studies were conducted to demonstrate that the data were reproducible and robust [7]. The methylation levels of three DMRs in normal tissue, adenoma, and CRC in the two data sets are shown in Figure 2. In CRCs or adenomas, all candidates had significantly higher methylation levels compared to normal tissues (*P* < 0.0001). However, there were no significant differences in methylation levels of the three candidate DMRs between CRCs and adenomas.

### 3.4. Performance of Selected Aberrant DNA Methylation as Potential Diagnostic Markers

**Figure 3:**
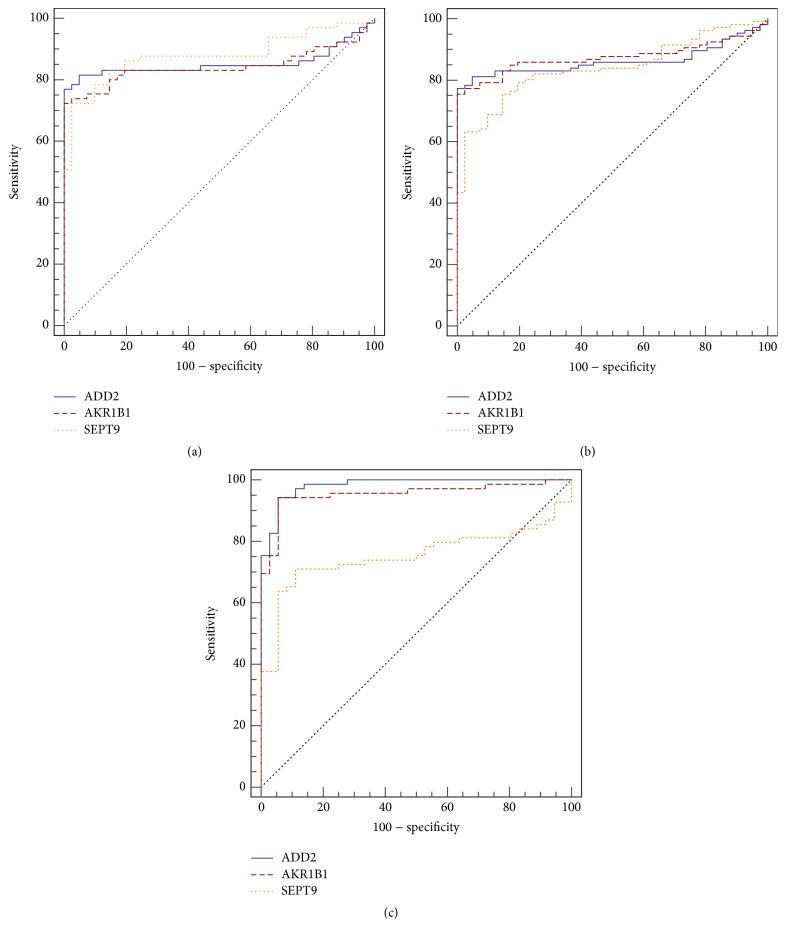
ROC curves of ADD2/AKR1B1/SEPT9 methylation using the data sets GSE48684 and GSE68060. (a) and (b) Receiver operating characteristic (ROC) curve showing accurate discrimination based on ADD2/AKR1B1/SEPT9 methylation using the data set GSE48684. (a) Between normal tissue and CRC tissue samples. (b) Between normal tissue and adenoma + CRC tissue samples. (c) ROC curve showing accurate discrimination based on ADD2/AKR1B1/SEPT9 methylation between normal tissue and CRC tissue samples using the data set GSE68060.

To determine whether these candidates could be potential biomarkers for use in diagnostics to distinguish between normal tissue and adenomas and CRC, we calculated AUC values for all three candidates in two data sets individually. In data set GSE48684, three markers had AUCs of 0.850 (*ADD2*, 95% CI: 0.767–0.912), 0.840 (*AKR1B1* 95% CI: 0.756–0.904), and 0.877 (*SEPT9*, 95% CI: 0.798–0.933) between normal tissues and CRCs (Figure 3(a)). The same three markers had AUCs of 0.862 (*ADD2*, 95% CI: 0.796–0.913), 0.874 (*AKR1B1*, 95% CI: 0.809–0.923), and 0.840 (*SEPT9*, 95% CI: 0.770–0.895) between normal tissues and adenomas + CRCs (Figure 3(b)). In data set GSE68060, the three markers showed AUCs of 0.982 (*ADD2*, 95% CI: 0.935–0.997), 0.954 (*AKR1B1* 95% CI: 0.895–0.985), and 0.752 (*SEPT9*, 95% CI: 0.659–0.831) between normal tissues and CRCs (Figure 3(c)). Two candidates,* ADD2* and* AKR1B1*, both showed better performances than* SEPT9*.

## 4. Discussion

Screening and early diagnosis is crucially important in the clinical management of CRC. Currently, colonoscopy and FOBT are the main approaches for CRC detection [8]. However, half of all CRCs are only detected at the advanced stages.

The widespread occurrence of modifications in CRC has major potential for being utilized as molecular markers, since alterations in DNA methylation in CRC was described by Fearon and Vogelstein over 20 years ago [9]. Compared with normal tissues, even adenomas showed apparent aberrant DNA methylation. Many aberrant DNA methylations have been reported as potential markers of CRC, such as* SEPT9*,* NDRG4*, and* VIM* [5, 10, 11]. To date, a blood-based assay named Epi proColon (Epigenomics AG, Berlin, Germany), which detects methylated* SEPT9*, has been applied clinically in several countries [12–14]. However, the sensitivity and specificity of* SEPT9* detection are still unsatisfactory. In a prospective clinical trial, sensitivity was 68% for all stages of CRC and 64% for CRC stages I–III, and much lower (22%) for advanced adenoma. In the present investigation, we performed a biomarker discovery and validation study to find new DNA methylation markers, which can be used for screening and diagnosing CRC.

Initially, we mapped the genome-wide methylation pattern of CRC compared with adjacent normal tissues using a 450 K bead chip and performed DMR analysis; this revealed that hypermethylation mainly occurred in CpG islands and promoter regions, while hypomethylation mainly occurred in the open sea and intergenic regions. These observations are in accordance with previous studies [15, 16]. We also found that most hypermethylated regions were short fragments (<400 bp), whereas most hypomethylated regions were long fragments (>1200 bp). These results suggest that hypermethylation occurs on a small scale and hypomethylation occurs on a large scale in CRC.

From the list of DMRs, we selected the candidates that most closely matched our criteria, which were set based on the premise that hypermethylation candidates are obviously better suited than hypomethylation ones for further clinical application. One of the candidates is* SEPT9*, which has already been applied clinically. The protein ADD2 is a subunit of adducin, a cytoskeletal protein, which caps and stabilizes the fast-growing end of actin filaments. ADD2 is usually expressed in the nervous system and erythroid tissues [17–19]. For the first time, the present study describes the hypermethylation in the promoter region of the* ADD2* gene in malignancy. The aberrant methylation of* AKR1B1* in CRC has been previously reported [20, 21]. However, the role of* AKR1B1* as a potential biomarker has not yet been demonstrated. Therefore, the two candidates (*ADD2*,* AKR1B1*) were compared with* SEPT9*, and the performances of* ADD2* and* AKR1B1* were further evaluated for their potential as biomarkers.

The Infinium Methylation 450 K bead array is the new generation of the Methylation 27 K bead array, which contains high density methylation probes with a distribution over the entire genome. Many investigations have demonstrated the accuracy and reproducibility of this technology and have shown that the results of the Infinium Methylation 450 K Bead Chip had a good positive correlation with bisulfite sequencing [22, 23]. In the present study, two independent data sets of the 450 K bead array from GEO were used for* in silico* validation. ROC curves were performed to determine the performance of the selected candidates. In the data set GSE48684,* ADD2* and* AKR1B1* have similar AUCs to* SEPT9* when CRCs are compared to normal tissues. Comparing adenoma + CRC with normal tissues,* ADD2* and* AKR1B1* have higher AUCs than* SEPT9*. When comparing CRC and normal tissues in GSE68060,* ADD2* and* AKR1B1* also have higher AUCs than* SEPT9*. These results suggest aberrant methylations of* ADD2* and* AKR1B1* may have better screening and diagnostic performances in the early detection of CRC than* SEPT9* alone. These findings should be confirmed by additional studies, for example, by carrying out tests in stools or blood.

## 5. Conclusions 

In summary, we conducted investigations into the discovery of tissue biomarkers to identify DNA methylation markers associated with CRC and then replicated the findings in two independent sets from GEO. Further studies are required to confirm these results and understand the role of these genes in colorectal carcinogenesis.

## Supplementary Material

Supplementary Materials include two tables. Clinicopathological characteristics of CRC patients are shown in Table S1, and the distribution of DMRs is shown in Table S2.

## Figures and Tables

**Table 1:** The list of top 10 DMRs by the *P* value.

Number	Start	End	Size	CHR	Arm	Gene	Feature	cgi	*P* value	Average deltaBeta
1	29520841	29521887	1047	6	p	NA	IGR	Shore	2.17*E* − 71	0.297
2	130130327	130132504	2178	7	q	MESTIT1	Body	Shore	4.20*E* − 63	0.118
3	33140275	33148582	8308	6	p	COL11A2	Body	Open sea	5.36*E* − 42	−0.147
4	33130824	33138475	7652	6	p	COL11A2	3′UTR	Shore	6.93*E* − 40	−0.134
5	27139876	27142774	2899	7	p	HOXA2	Body	Shelf	3.18*E* − 35	0.232
6	133561800	133562545	746	6	q	EYA4	TSS1500	Shore	2.09*E* − 34	0.306
7	31938678	31939388	711	6	p	STK19	TSS1500	Shore	9.84*E* − 32	−0.149
8	32183994	32190096	6103	6	p	NOTCH4	Body	Open sea	7.50*E* − 31	−0.151
9	78493049	78493778	730	13	q	EDNRB	5′UTR	Island	9.47*E* − 31	0.296
10	30078146	30080891	2746	6	p	TRIM31	Body	Open sea	1.47*E* − 26	−0.196

**Table 2:** The list of candidate DMRs for validation.

Number	Start	End	Size	CHR	Arm	Gene	Feature	cgi	*P* value	Average deltaBeta
1	70995426	70995462	37	2	p	ADD2	TSS200	Shore	2.22*E* − 08	0.41
2	75369210	75369237	28	17	q	SEPT9	TSS200	Island	3.74*E* − 05	0.41
3	134144040	134144184	145	7	q	AKR1B1	TSS200	Island	1.09*E* − 07	0.41

## Abbreviations

AUC: Area under curve
CRC: Colorectal carcinoma
DMR: Differentially methylated region
FOBT: Fecal occult blood testing
GEO: Gene Expression Omnibus
MSP: Methylation-Specific Polymerase Chain Reaction
ROC: Receiver operating characteristic
UTR: Untranslated Regions.
