# Supplementary material for: Discovery and Validation of Hypermethylated Markers for Colorectal Cancer
Source: Dis Markers. 2016 Jul 14;2016:2192853. doi: 10.1155/2016/2192853 (PMC4963574; doi:10.1155/2016/2192853)
Supplement: Supplementary file 1 — Supplementary Materials include two tables. Clinicopathological characteristics of CRC patients are shown in Table S1, and the distribution of DMRs is shown in Table S2. [file 2192853.f1.docx]

**Table S1 *Clinicopathological characteristics of colorectal cancer patients***

|  | Patient 1 | Patient 2 | Patient 3 | Patient 4 | Patient 5 | Patient 6 |
| --- | --- | --- | --- | --- | --- | --- |
| Gender | female | male | female | female | female | male |
| Age | 76 | 50 | 63 | 68 | 73 | 63 |
| Differentiation | moderate | moderate | moderate | poor | moderate | moderate |
| Location | rectum | rectum | rectum | rectum | rectum | rectum |
| Stage | II | II | II | III | III | III |
